# Epilepsy

**Published:** 1886-08-20

**Authors:** C. L. Dana

**Affiliations:** New York


### EPILEPSY.

BY C. L. DANA, M.D., OF NEW YORK.

If one may be permitted to be aphoristic on subjects like this, I would express my views regarding the therapeutics of epilesy as follows :

I. Diet, exercise, and proper hygienic treatment, including baths, rank above all other single therapeutic measures.

If I had to choose between them and bromides even, I would select the former. On the whole, I fear that the reign of bromides has made the condition of epileptics more miserable than it was before, and has done our patients an actual harm. At the best, not more than 5 or 10 per cent. of epileptics can be cured, while the majority are reduced by bromides to a state of physical enfeeblement and distressing mental hebetude. Besides, physicians now fly to the bromides, and, trusting to them, neglect the searching inquiry into the exciting cause or the careful direction of the patient's habits, which are so essential.

All medical experience unites in ascribing benefit to judicious regulation of diet and exercise. The only difference of late years is that we do not now believe epileptics should be starved.

Epileptics should live on plain, easily digestible diet, containing a preponderance of fat. A special meat diet, or milk diet, or farinaceous diet, is not injurious nor curative. It all depends upon the patient. A meat diet may be best if the patient is lithæmic or has a fermentative dyspepsia. A milk diet is especially useful for children and erythritic women. The meals should be small, and, in case of voracious appetite, four, five, or six light meals a day should be given. No heavy meal should be taken within twenty-four hours of sleeping. Indeed, no heavy meals should ever be taken by epileptics. Special diets have often to be rigidly laid down simply as a matter of discipline, since patients will not follow any general directions. I believe that the urine and digestive functions should be carefully studied, and that thus we shall find indications for selecting the right kind of food.

II. The bromides take the second rank in the treatment of epilepsy.

All bromides act alike in this disease. If one does not cure, another will not. Occasionally changing and mixing reduces the attacks for a time, and benefits the stomach.

III. The best bromides are those of potassium, sodium, ammonium, and hydrogen (hydro-bromic acid) ; possibly we may add nickel.

Bromide of potassium is the most trustworthy.

Bromide of sodium is more agreeable to the taste, less irritating to the stomach, and milder in its effects, but is eventually just as depressing as other forms.

Bromide of ammonium has a brief stimulant effect on the circulation.

Hydrobromic acid is useful in those cases in which there are indigestion and phosphaturia, and an alkali is contra-indicated. It produces acne less readily than the alkaline bromides.

IV. Bromides should be given in daily doses of ʒ j, increased gradually until the attacks are suppressed, or the dose reaches ʒ iv to ℥ j daily. Few patients can tolerate more than this latter dose. Thorough bromidization should be always tried if necessary to stop the fits, and it may be occasionally repeated. But bromidzation is sometimes injurious, even making the disease worse, and it must always be employed with caution.

V. When the fits are suppressed the bromides should be carefully reduced, but never entirely stopped for at least two years after the last fit.

VI. In most cases, and especially in nocturnal epilepsy, an extra large dose of bromide should be given at night.

VII. It is very important that bromides should be chemically pure, and their use should be continued a very long time, and that their depressing effects should be offset by tonics and all possible roborant measures.

VIII. The best non-specific adjuvants (drugs) to the bromides are potassium iodide (in syphilitic epilepsy), potassium bicarbonate (in lithæmic and rheumatic states), carbonate of ammonium, the hypophosphites, arsenic, iron, and quinine.

IX. The other chief adjuvants to the bromides are diet, exercise, a regular life, hydrotherapy, counter-irritation on the neck, and, in the line of drugs, zinc, belladonna, strychnine, valerian, and the nitrite. Combinations of bromides with the other drugs mentioned will lessen attacks when bromides alone will not.

Other drugs which sometimes help the bromides are digitalis, *Cannabis indica*, ergot, conium, chloral, the salts ot copper, picrotoxin, and borax. None of these do any permanent good alone, and their value as adjuvants is not uniform or generally conceded.

X. The best substitute for the bromides when these do no good, or do harm, are belladonna, zinc, strychnine, glonoin, borax, and alteratives.

XI. Bromides stop the fits in from 5 to 10 per cent. of cases, oftener if given early in the disease, if given to young children, and if given in cases that develop after twenty-one.

Bromides lessen the fits in from 80 to 85 per cent. of cases.

Bromides do no good, or do actual harm, as regards frequency of attacks, in from 5 to 10 per cent. of cases.

Bromides do no actual good to the patient in a much larger proportion ot cases.

XII. To prevent bromide acne, arsenic, calcium sulphide, baths, and diuretics are the best measures, or hydrobromic acid may be used.

To prevent bromidization, adopt all possible roborant measures; use salt-water baths and regular physical exercise ; give black coffee, caffeine, cocaine, mineral acids, strychnine, bitter tonics, cod-liver oil, or give large doses of the bromides every three days only.

In all cases dilute the bromide, preferably with carbonic acid water or Vichy, in the proportion of six ounces of water to a scruple of the drug.

The continuous administration of an alkaline bromide in an alkaline water sometimes affects the bladder, and then the bromides can be given dissolved in hydrobromic acid.

XIII. The remedies that are especially useful in *petit mal* are, after the bromides, belladonna, glonoin (?), Cannabis indica (?), cod-liver oil, ergot (?), counter-irritation at the back of the neck, and cold spinal douches

XIV. For epilepsy in children, besides the bromides, it is advisable to employ a milk diet, rest, and oxide of zinc. Belladonna, if tried, should be given cautiously.

XV. For adults and chronic cases, use the bromides, belladonna, iodide of potassium, and ammoniated sulphate of copper. Oxide of zinc is here of less value.

XVI. For nocturnal epilepsy, increase the dose of bromide at night, and add chloral or digitalis. Give also, if needed, strychnine. Raising the head of the bed or making the patient sleep in a chair at night, are measures to be tried.

XVII. For hysterical and erythritic cases, with, or in place of, bromides, give a diet of vegetables. Try turpentine, valerian, or zinc. Belladonna is usually contra-indicated.

XVIII. Counter-irritation by means of blisters, issues, and setons at the back of the neck, is a useful adjunct to treatment, especially in *petit mal* and in cases with mental derangement.

XIX. For the status epilepticus, give large enemata of chloral, and use emetics and purges. Venesection is often efficacious; morphine is dangerous; chloroform is only palliative, and nitrite of amyl is of little value.

XX. To prevent impending attacks, the best remedy is nitrite of amyl, which may be carried in a vial filled with cotton. Inhalation of chloroform or ammonia, the internal administration of ammonia, spirits of lavender or alcohol, a sternutatory, and pressure on the carotids—all are measures which sometimes stop the attack.

XXI. Alterative and habit-breaking drugs, such as mercury, iodide of potassium, arsenic, antimony, are useful in epilepsy.

XXII. No surgical measures upon ovaries, uterine, testicles, cranium, or elsewhere will cure an established, long-standing epilepsy, except in rare cases. Such operations, if done, should be undertaken early, before the patient has had an excessive number of fits.—*N. Y. Medical Journal.*

